# Handling hybrid and missing data in constraint-based causal discovery to study the etiology of ADHD 

**DOI:** 10.1007/s41060-016-0034-x

**Published:** 2016-12-02

**Authors:** Elena Sokolova, Daniel von Rhein, Jilly Naaijen, Perry Groot, Tom Claassen, Jan Buitelaar, Tom Heskes

**Affiliations:** 1 Faculty of Science, Radboud University Nijmegen, Nijmegen, The Netherlands; 2 Donders Institute for Brain, Cognition and Behaviour, Radboud University Medical Center Nijmegen, Nijmegen, The Netherlands

**Keywords:** Causal discovery, Missing data, Mixture of discrete and continuous data, ADHD

## Abstract

Causal discovery is an increasingly important method for data analysis in the field of medical research. In this paper, we consider two challenges in causal discovery that occur very often when working with medical data: a mixture of discrete and continuous variables and a substantial amount of missing values. To the best of our knowledge, there are no methods that can handle both challenges at the same time. In this paper, we develop a new method that can handle these challenges based on the assumption that data are missing at random and that continuous variables obey a non-paranormal distribution. We demonstrate the validity of our approach for causal discovery on simulated data as well as on two real-world data sets from a monetary incentive delay task and a reversal learning task. Our results help in the understanding of the etiology of attention-deficit/hyperactivity disorder (ADHD).

**Electronic supplementary material:**

The online version of this article (doi:10.1007/s41060-016-0034-x) contains supplementary material, which is available to authorized users.

## Introduction

In recent years, the use of causal discovery in the field of medical research has become increasingly popular. Causal discovery analyses all variables together and suggests causal dependencies between variables, providing better insight into the data. This approach has several advantages in comparison with standard statistical techniques. First, causal discovery provides an opportunity to learn causes and effects from the observed data, without performing experiments that can be costly and time-consuming. Second, it detects whether the dependency between variables is direct or mediated through other variables. Third, it can visualize the results in the form of a graph that makes the results easier to interpret.

Even though there are a variety of algorithms that can learn the structure of the causal network for medical data, there are still many challenges in this field of research. In this paper, we discuss two of them. The first challenge is dealing with data that contains a mixture of discrete and continuous variables. Medical data often contain both discrete and continuous variables, where continuous variables are not necessarily normally distributed. The second challenge is dealing with incomplete data. In practice, some tests are performed only for part of the patients, the quality of some data is poor, participants drop out, etc.

Although there are methods that can handle mixed variables or missing values separately, to the best of our knowledge there is no algorithm that can handle both challenges simultaneously for directed graphical models. However, there are such methods for undirected graphical models. In Refs. [1, 34, 53], the authors propose different methods to estimate the correlation matrix for data with missing values and mixture variables, and based on this correlation matrix learn the structure of the undirected graphical model.

Algorithms that search for a structure of directed and undirected graphical models have a lot in common. They both try to find the optimal structure that provides the lowest complexity and the best goodness of fit. The main difference is that one model gives as output a directed graph and another gives an undirected graph. In this paper, we propose to transfer the ideas of structure learning for undirected graphical models to causal discovery.

We propose a method that can handle missing values and mixture variables based on the ideas for undirected graphical models presented in Refs. [1, 53]. This method relies on two main assumptions. The first assumption is that the part of the data with continuous variables obeys a so-called non-paranormal distribution. For univariate monotone functions ${f_1, \ldots , f_d}$ and a positive definite correlation matrix $\mathrm {\Sigma ^0}\in {\mathbb {R}}^{d\times d}$, we say that a $d$-dimensional random variable ${ X= (X_1,\ldots , X_d)^T}$ has a non-paranormal distribution $X\backsim \mathrm {NPN}_d(f, \mathrm {\Sigma ^0})$, if ${f(X)= (f_1(X_1),\ldots ,f_d(X_d))} \backsim N_d(\mathrm {0}, \mathrm {\Sigma ^0})$. We further assume functions $f_1,\ldots ,f_d$ to be strictly monotone enabling computational tractability of the non-paranormal. A non-paranormal distribution implies that observed variables have monotonic relationships. This comes from the fact that a Gaussian distribution implies linear, hence monotonic relationships between the surrogate variables $f_i(X_i)$. Moreover, the monotonic relationship from surrogate Gaussian variables $f_i(X_i)$ to observed variables $X_i$ does not change their ratings. That implies monotonic relationships between observed variables as well. For most real-world medical data, this is a reasonable assumption, since medical data usually has a relatively small sample size and non-monotonic dependencies, if present, are difficult to detect. The second assumption is that data are missing at random (MAR). This is also a reasonable assumption for many medical studies where the missing data often occur due to the fact that some experiments finish faster than others. As a result, information about symptoms, age, gender is usually present for all patients at the beginning of the study, while information about genes or brain functioning takes years to be collected and then may be missing for some subjects.

We propose a three-step algorithm: (1) Transform initial data into a Gaussian distribution by transforming the data first to the empirical distribution and then to Gaussian normal scores. This step deals with a mixture of discrete and continuous variables with non-paranormal distribution. (2) Use the expectation maximization (EM) algorithm to estimate the correlation matrix for this data. This step deals with missing values. (3) Apply a causal discovery algorithm to learn the causal structure from the correlation matrix. In this paper, we use the Bayesian Constraint-based Causal Discovery (BCCD) algorithm [10] which is a state-of-the-art algorithm for causal discovery. This step outputs the causal graph and provides a reliability measure for each edge in the graph.

In the first part of the algorithm, we use a copula transformation to estimate the correlation between variables. This approach has been shown to work well for variables with non-paranormal distributions [22, 53]. In our case, we apply the same approach for a mixture of discrete and continuous variables and model the distribution of both discrete and continuous variables using a Gaussian copula to obtain an approximation of the correlation matrix. This leads to a slight underestimation of some correlations [26]. In case the focus of the research is the causal directed acyclic graph (DAG) from the observed variables, conditional independencies involving discrete variables do not exactly correspond to conditional independencies between their surrogate Gaussian variables. In this paper, we focus on independencies in the surrogate variables and assume that our data comes from a causal DAG in the latent space. Following Abegaz and Wit [1], i.e., it might not be necessary to use complex methods to model discrete variables, since this would not result in a significant increase in accuracy. Further in the simulation study, we demonstrate that using this approximation our algorithm manages to accurately estimate the causal structure.

We compare the first two steps of the proposed algorithm with alternative methods. For the first step instead of transforming data to a Gaussian we transform it to ranks. For the second step instead of EM we use pairwise correlation, list-wise deletion, and mean imputation. Although these methods rely on a stronger assumption than EM that data are missing completely at random (MCAR), we choose them as a common alternative to EM. We show that EM with Gaussian transformation performs better than the alternative methods, when the amount of missing data is significant. We also show that the strength of the dependencies in the data influences the method that should be used to estimate the correlation matrix for causal discovery. Thus, even though the methods that are considered in this paper to estimate correlation matrices have similar performance for the undirected graphical model, our analysis suggests that these methods have a different effect on the accuracy of a causal discovery algorithm. To test the validity of our conclusions that EM with a Gaussian transformation performs better than alternatives for directed graphical models, we repeat the same experiments with the PC algorithm instead of BCCD.

As a prototypical example, we apply the proposed algorithm to two data sets about attention-deficit/hyperactivity disorder (ADHD). ADHD is a frequent and highly heritable neuropsychiatric disorder, affecting 5–6% of children [41]. Symptoms persist into adulthood in up to 50% of the childhood cases [17]. ADHD is characterized by two types of symptoms: hyperactivity/impulsivity and inattention, which can occur separately or combined. Given the large number of patients and long term impact of the disorder on patients and health care system, ADHD is a serious financial burden to society.

Both ADHD data sets used in this paper have all features of a typical medical data set since they describe causal relationships between various possible factors of the disease such as genes, age, gender, and different types of symptoms and behavioral characteristics. These data sets have several possible factors, which can influence symptoms and interact with each other. The first data set describing a monetary incentive delay task has a moderate sample size of 409 subjects and approximately 10% of missing data. The second data set describing a reversal task has a sample size of 271 subjects and 0.3% of missing data. Both data sets have a mixture of discrete and continuous variables.

These data sets are part of the NeuroIMAGE project (see www.neuroimage.nl), whose goal is to learn cognitive, neural (MRI, MRS), and genetic underpinnings of ADHD. The first data set [51] investigates the role of the genetic factors on the ADHD symptoms, and brain functioning measured during the reward related task. The second data set studies how problems with learning from reinforcement are associated with ADHD symptoms using a probabilistic reversal learning task (PRL). Based on this data, we build two causal models that provide deeper understanding of the altered reward processing and reversal learning in adolescents with ADHD than standard statistical tests. These models can help to understand the mechanisms that drive ADHD and make treatment more effective.

Parts of this work have been published as conference papers [47, 48]. In Ref. [47], we proposed an approach for causal discovery from data with a mixture of discrete and continuous variables. We focus on the use of the mutual information for scoring structures and explore the quality of our estimates for the mutual information through simulation studies. We applied our approach to real-world data from the ADHD-200 competition. In the short paper [48], we described the extension of the method in Ref. [47] to handle missing values and demonstrate it on a data set involving a monetary incentive delay (MID) task. In the current paper, we describe our approach in much more detail and extend our previous work in a number of directions: (1) We provide an extensive simulation study where we compare our method with alternative approaches; (2) we describe the application of our method on a new data set involving a reversal learning task; and (3) extend its application on the MID task data set with a detailed interpretation of the results provided by a medical expert.

The rest of the paper is organized as follows. Section 2 describes background information about causal discovery and graphical models. Section 3 describes algorithms for structure learning. Section 4 explains the proposed method. Section 5 presents the results of the experiments on simulated data and ADHD data. Section 6 provides our conclusion and future work.

## Background

A Bayesian network is a pair $$({\mathcal {G}},\varvec{\varTheta })$$ where $${\mathcal {G}}= ({\mathbf {X}}, {\mathbf {E}})$$ is a DAG with a set of nodes $${\mathbf {X}}$$ representing domain variables and a set of arcs $${\mathbf {E}}$$; $$ \theta _{X_i}\subset \varvec{\varTheta }$$ is a set of parameters representing the conditional probability of variable $$ X_i \subset {\mathbf {X}}$$ given its parents $$ Pa _{i}$$ in a graph $${\mathcal {G}}$$. Using Bayesian networks, we can model causal relationships between variables. In that case, an edge $$ A \rightarrow B $$ between variables represents a direct causal link from $$ A $$ to $$ B $$. This means that $$ A $$ influences the values of $$ B $$, but not the other way around.

Saying that two variables $A$ and $B$ are conditionally independent given $C$, means that if we know $C$, learning $B$ would not change our belief in $A$. Two DAGs are called equivalent to one another, if they entail the same conditional (in)dependencies. All DAGs that are equivalent to a graph ${\mathcal {G}}$ form an equivalence class of a graph ${\mathcal {G}}$, where all members are indistinguishable in terms of implied independencies. To represent the members of this equivalence class, a different type of structure is used, known as a partially directed acyclic graph (PDAG).

The three main assumptions that are often used when learning the structure of causal networks are the following [49]:

1. Causal Markov Condition: each variable is independent of its non-descendant conditioned on all its direct causes.
2. Faithfulness assumption: there are no independencies between variables that are not implied by the Causal Markov Condition.
3. Causal sufficiency assumption: there are no common confounders of the observed variables in ${\mathcal {G}}$ that are not members of the set.

In this paper, we do not rely on the causal sufficiency assumption, i.e., we do allow for latent variables. One can represent the structure of a Bayesian network with latent variables using a so-called Maximal Ancestral Graph (MAG) [43] on only the observed variables. In contrast to DAGs, MAGs can also contain bi-directed $X \leftrightarrow Y$ arcs (indicating that there is a common confounder) and undirected arcs $X - Y$ (meaning that there is a selection bias affecting $X$ and $Y$). The equivalence class for MAGs can be represented by a partial ancestral graph (PAG) [55]. Edge directions are marked with “ − ” and “>” if the direction is the same for all MAGs corresponding to the PAG and with “$\circ$” otherwise.

## Related study and motivation

In this section, we discuss existing methods for causal discovery. Since there are no algorithms that can handle mixture variables and missing data simultaneously, we consider the methods that can handle at least one of the challenges. Then we discuss how both challenges are solved for undirected graphical models and in Sect. 4 propose how can we transfer these ideas to directed models.

### Structure learning

Causal discovery requires structure learning for directed graphical models. There are many methods that can be used to learn the structure of directed graphical models. A broad description of methods can be found in Ref. [11]. In general, methods are divided into two approaches: constraint-based and score-based. The constraint-based approach works with statistical independence tests. First, this approach finds a skeleton of a graph by starting from the complete graph and excludes edges between variables that are conditionally independent, given some other set of variables (possibly empty). Second, the edges are oriented to arrive at an output graph. The constraint-based approach learns the equivalence class of DAGs and outputs a PDAG. Examples of the constraint-based approach are the IC algorithm [38], PC-FCI [49], and TC [39]. The score-based approach uses a scoring metric. It measures the data goodness of fit given a particular graph structure and accounts for the complexity of the network. There are many different scoring metrics, where the Bayesian score [12] and the BIC score [46] are among the most common. The goal is to find the graph that has the highest score. Unfortunately, this optimization problem is NP-hard, so different heuristics are used in practice. These methods are divided in local search methods, such as greedy search [9], greedy equivalence search [8], and global search methods, such as simulated annealing [13] and genetic algorithms [31].

An advantage of the constraint-based approach is that it does not have to rely on the causal sufficiency assumption, which means that the algorithm can detect common causes of the observed variables. A disadvantage of the constraint-based approach is that it is sensitive to propagating mistakes in the resulting graph. A standard approach makes use of independence tests, which results for borderline independencies/dependencies sometimes can be incorrect. The outcome of learning a network can be sensitive to such errors. In particular, one such error can produce multiple errors in the resulting graph. A set of conservative methods such as conservative PC (CPC) [42] and conservative FCI (CFCI) [50] tackles the problem of lack of robustness, outperforming standard constraint-based methods such as PC. An advantage of the score-based approach is that it provides a measure of reliability of inferred causal relations. This makes the interpretation of the results easier and prevents incorrect categorical decisions. A main drawback of the approach is that it relies on the causal sufficiency assumption and as a result cannot detect latent confounders.

To deal with a mixture of discrete and continuous variables, several methods have been proposed for constraint-based structure learning. Spirtes et al. [49] proposed to use conditional independence tests based on partial correlation. Harris and Drton [22] showed that substituting Pearson correlation by Spearman correlation, the PC algorithm is able to infer a correct network structure under the assumption that data obey a Gaussian copula distribution. Margaritis [35] developed a conditional independence test that does not rely on the distribution of the variables, but the test still involves discretization of the variables. Several methods have been proposed for score-based methods that can work with a mixture of discrete and continuous variables. Geiger and Heckerman [21] proposed a closed-form solution for the Bayesian score of a mixture of discrete and continuous variables, but this solution only works in case a number of assumptions are met. These assumptions imply that the data are drawn from a conditional Gaussian distribution and forbid structures in the network with a continuous variable having a discrete variable as a child. An alternative method is described in Ref. [15] which uses a multiple regression framework for scoring structures. However, the method is applicable only for time-series data. Bach and Jordan [3] use Mercer kernels to estimate the structure of causal models, but calculation of a Gramm matrix requires significant computational costs ($O(N^3)$, where *N* is the sample size) and may be inefficient for data sets with large sample sizes. Monti and Cooper [36] use neural networks to represent the density function for a mixture of discrete and continuous variables. Estimation of the neural network parameters requires significant computational costs which makes this approach computationally expensive.

To deal with missing values, several methods have been proposed to learn the structure of the network in the presence of missing values. Friedman [20] proposed a Structural EM algorithm to estimate a Bayesian network that has been further developed by Bernardo et al. [6]. The disadvantage of the EM algorithm is that it can get stuck in a local minimum. To prevent this, an evolutionary algorithm in combination with MCMC was proposed in Ref. [44]. The limitation of these algorithms is that they usually rely on the assumption that data are either discrete or continuous Gaussian.

### Undirected graphical models

Undirected graphical models build a graph where nodes represent variables and edges describe conditional independence relationships between the variables. The conditional independence relationships are estimated using the precision matrix (the inverse of a covariance matrix). Assuming that the precision matrix is sparse, the sparseness constraint is incorporated in the estimation of the precision matrix. That results in an optimization problem [4] to find the inverse correlation matrix $\varTheta =\Sigma ^{-1}$ with the best combination of goodness of fit and sparsity:

1 $$\begin{aligned} \max f(\varTheta )= \log \det \varTheta -\text {tr}(S\varTheta )-\lambda \Vert \varTheta \Vert _1. \end{aligned}$$

Here tr denotes the matrix trace, det denotes determinant, $\Vert \varTheta \Vert _1$ denotes the $L_1$ norm, *S* denotes the empirical covariance matrix, and $\lambda >0$ is a regularization parameter. In some sense, score-based structure learning algorithms for directed graphical models solve a similar optimization problem, but produce a directed graph as output.

In recent years, considerable effort has been invested in estimating the structure of undirected graphical models for non-Gaussian data and data containing missing values [1, 53]. The precision matrix can be estimated under the assumption that data obey a non-paranormal distribution. In that case, Pearson correlation, which relies on the assumption of Gaussian data, is substituted by Spearman (Rho) rank correlation ($\rho$) [1, 34, 53]. An adjustment to the final Spearman’s rho correlation is applied in order to make it close to the Pearson correlation matrix, when the data are indeed Gaussian [28, 30]:

2 $$\begin{aligned} S= 2\sin (\pi \rho /6). \end{aligned}$$

The precision matrix can still be estimated when there are missing values in the data. One can use pairwise analysis and calculate pairwise correlation instead of complete case correlation to estimate the matrix [1, 53]. As a result, one can keep as much data as possible. Another advantage of the pairwise correlation is that it does not introduce any bias to the results in contrast to imputation methods. However, there is no guarantee that the correlation matrix will be positive definite when we use pairwise correlation for data with missing values. In that case, a projection to the closest positive definite correlation matrix can be made [7, 23].

Alternatively, the expectation maximization (EM) algorithm can be used to estimate the values of the correlation matrix $\Sigma$ [14, 33]. The EM algorithm requires Gaussian data, so a copula transformation to Gaussian data can be used. The EM algorithm guarantees that the matrix would be positive definite, so no further adjustments are required.

Using Spearman pairwise correlation or the EM algorithm in combination with an optimization subroutine like Glasso or DoPing showed to be one of the best methods in the field of undirected graphical models to estimate the structure of the graph with data obeying a non-paranormal distribution and missing values [53]. In this paper, we transfer these ideas to learn the structure of a causal graph and compare different methods using simulated and real-world data.

## Proposed method

In this section, we propose a causal discovery algorithm that can deal with both a mixture of discrete and continuous variables as well as missing data. In the first two steps of this algorithm, we estimate the correlation matrix, when the data has mixture variables and missing data, based on the ideas described in Sect. 3. In the third step, we use this correlation matrix as an input into a causal discovery algorithm to infer the causal structure. We use the BCCD algorithm for this purpose, one of the state-of-the-art algorithms in causal discovery. Claassen and Heskes [10] showed that BCCD outperforms reference algorithms in the field, such as FCI and Conservative PC. Moreover, it provides an indication of the reliability of the causal links that makes it easier to interpret the results and compare alternative models. The advantage of the BCCD algorithm is that it combines the strength of constraint-based and score-based approaches. We rely on the assumption that data are missing at random and that continuous variables obey a non-paranormal distribution.

We propose the following algorithm:

- Step 1: Mixture of discrete and continuous variables
  To deal with data sets that contain a mixture of discrete and continuous variables, we propose to use a Gaussian copula. For each variable $X_i$ in the data set, we estimate the rescaled empirical distribution
  3 $$\begin{aligned} {\hat{F}}_i(x)=\frac{1}{n+1}\sum _{j=1}^{n}{\mathcal {I}}\{X_{i,j}<x\}, \end{aligned}$$
  where ${\mathcal {I}}$ is an indicator function and then transform the data into Gaussian normal scores
  4 $$\begin{aligned} {\hat{X}}_i={\hat{\varPhi }}_i^{-1}({\hat{F}}(X_i)). \end{aligned}$$
  In this step missing values are ignored.
- Step 2: Correlation matrix with missing data
  The next step is to estimate the correlation between the variables in the model. This correlation matrix will be used in the next steps, where we will estimate the causal structure of the graph. New variables now have a Gaussian distribution, so we can use Pearson correlation to estimate dependencies between variables. Since our data has missing values, we propose to first use the EM algorithm to estimate the correlation matrix, since this algorithm provides an unbiased estimate of parameters and their standard error [14].
  The EM algorithm searches for the Maximum Likelihood Estimate (MLE) of the marginal likelihood by iteratively applying the following two steps:
  1. E-step: Estimate the sufficient statistics;
  2. M-step: Re-estimate the covariance matrix using the sufficient statistics from the previous step. Re-estimate missing values. The algorithm iterates until convergence.
  The output of EM is a covariance matrix that should be normalized to have unit variance.
- Step 3: Apply BCCD
  The correlation matrix is used in the BCCD algorithm to estimate the causal structure of the graph. We here describe only the basic idea of the BCCD algorithm. A more detailed description can be found in Ref. [10]. The BCCD algorithm contains two main steps:
*Step 3.1* Start with a fully connected graph and perform adjacency search, estimating the reliability of causal relations, for example, $X \rightarrow Y$. If a causal relation declares that variables are conditionally independent with a reliability higher than a predefined threshold, delete an edge from the graph between these variables. To estimate the reliability of the causal statement, we have to do the following substeps repeatedly:
  - First we estimate the mutual information, using the correlation matrix $\Sigma$ that we get as an output from Step 2. We propose to use the following formula:
    5 $$\begin{aligned} I(X_i, X_{\mathrm{Pa}_i})=-\frac{1}{2}\log \frac{|\Sigma _{i,\mathrm{Pa}_i}\mid }{\mid \Sigma _{\mathrm{Pa}_i}\mid }, \end{aligned}$$
    where $X_{\mathrm{Pa}_i}$ are the parents of node $i$ in DAG ${\mathcal {G}}$, $\Sigma _{\mathrm{Pa}_i}$ is a correlation matrix between the parents of variable $X_i$, and $\Sigma _{i,\mathrm{Pa}_i}$ is a correlation matrix between variable $X_i$ and its parents.
  - Knowing the value of mutual information, we can estimate the Bayesian Information Criterion (BIC) for data ${\mathbf{D}}$ that can then be used to compare scores of different DAGs (${\mathcal {G}}$). The BIC score is decomposed into the sum of two components, the mutual information $I ( X _ i , X_{\mathrm{Pa}_i} )$ estimated in the previous substep and $Dim[{\mathcal {G}}]$ the number of parameters necessary to estimate the model.
    6 $$\begin{aligned} \hbox {BIC\,score} ({\mathbf{D}}|{\mathcal {G}})&=M\sum _{i=1}^{n} I ( X _ i , X_{\mathrm{Pa}_i} )\nonumber \\&\quad -\frac{\log M}{2}\hbox {Dim}[{\mathcal {G}}], \end{aligned}$$
    where $n$ is the number of variables, and $M$ is the sample size. The first component measures the goodness of fit, and the second penalizes the complexity of the model.
  - To estimate the reliability measure, we need to estimate the marginal likelihood $p({\mathbf{D}}|{\mathcal {G}})$. We propose to use BIC, which approximates the logarithm of the marginal likelihood:
    7 $$\begin{aligned} \log p({\mathbf{D}}|{\mathcal {G}})= \hbox {BIC\,score} + O(1). \end{aligned}$$
    To get the probability $p({\mathbf{D}}|{\mathcal {G}})$, we should calculate (7) for all possible graphs for this subset of variables and then normalize it.
  - Now we can estimate the reliability of the causal statement *L*, e.g., $L:`X\rightarrow Y$’. It gives a conservative estimate of the probability of a causal relation. We estimate the reliability measure using a Bayesian score:
    8 $$\begin{aligned} p(L| {\mathbf{D}}) =\frac{\sum _{{\mathcal {M}}\in {\mathbf{M}}(L)}p({\mathbf{D}}|{\mathcal {M}})p({\mathcal {M}})}{\sum _{{\mathcal {M}}\in {\mathbf{M}}}p({\mathbf{D}}|{\mathcal {M}})p({\mathcal {M}})}, \end{aligned}$$
    where $p({\mathbf{D}}|{\mathcal {M}})$ denotes the probability of data ${\mathbf {D}}$ given structure ${\mathcal {M}}$, $p({\mathcal {M}})$ represents the prior distribution over structures, and ${\mathbf{M}}(L)$ is the set of structures containing the relation $L$. In this equation, we approximate the probability $p({\mathbf{D}}|{\mathcal {M}})$ by $p({\mathbf{D}}|{\mathcal {G}})$, which was calculated in the previous substep. Equation (8) also requires to set the prior distribution for $p({\mathcal {M}})$. Claassen and Heskes [10] propose to use a uniform prior.
*Step 3.2* Rank all causal relations in decreasing order of reliability and orient edges in the graph starting from the most reliable relations. If there is a conflict, pick the causal relation that has a higher reliability.

**Fig. 1:**
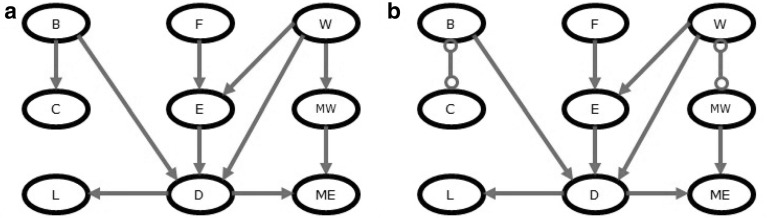
Waste Incinerator Network represented as **a** DAG, and **b** PAG. The node names are abbreviated as follows: Burning regime (*B*), Filter state (*F*), Waste type (*W*), CO$_2$ concentration (*C*), Filter efficiency (*E*), Metal in waste (*MW*), Light penetrability (*L*), Dust emission (*D*), Metals emission (*ME*)

To estimate Eq. (8) in Step 3.1, the algorithm requires calculating the marginal likelihood over all possible graphs for each causal relation that we infer. For speed and efficiency of the algorithm, the set of possible graphs is limited to the graphs with at most five vertices, which gives a list of at most 29,281 DAGs per set of five variables [10] to reduce the computational complexity. In theory, limiting the number of vertices to five may lead to a loss of information. In practice, however, the accuracy of the BCCD algorithm is hardly affected and it still outperforms standard algorithms that perform conditional independence tests for more than five variables [10].

In our method, we assume that each observed variable has a corresponding latent, surrogate variable, with a monotonic relationship between the two. The latent variable can thus be seen as a surrogate value, representing the exact same concept as the corresponding observed variable. The method infers and then depicts in the output graph the causal structure between these surrogate variables.

Each step in the proposed algorithm has several possible alternative solutions. Based on the papers about undirected graphical models [1, 53], an alternative for Step 1 is to transform data to ranks and use Spearman to deal with mixture variables. To deal with missing variables in Step 2, we use either pairwise correlation, mean imputation, or list-wise deletion to deal with missing values. In case of pairwise correlation, there are no guarantees that the correlation matrix will be positive definite and if not it should be projected to the closest positive definite matrix. Calculating Spearman pairwise correlation we have two alternatives: to apply the transformation proposed in Eq. (2) or not to apply.

An alternative to Step 3 could be any score-based causal discovery algorithm that can use a correlation matrix as an input. In this paper, we focus on the alternatives for Steps 1 and 2 and would like to learn which approach is the best for directed graphical models. Thus, we do not try to find the best alternative for Step 3, but rather check whether the best approach for Step 1 and 2 is the same when we use a different causal discovery algorithm. In order to do so, we compare our results with the PC algorithm.

## Experimental results

### Simulation study

To estimate the accuracy of the causal discovery for different alternatives of Steps 1 and 2 of the algorithm discussed in the previous section, we made a simulation study. We chose the Waste Incinerator Network [32] which contains a mixture of discrete and continuous variables. The Waste Incinerator Network describes the emission from a waste incinerator depending on the filter efficiency, waste type, burning regime, and other factors. The network contains nine variables that are connected by ten arcs as can be seen in Fig. 1.

The original version of the network contains continuous Gaussian variables. To make these variables nonnormal, we applied a monotonic transformation ($X^3$). We considered the Waste Incinerator Network when the correlation between variables is extreme-high (the correlation matrix is close to singular) and medium (the parameters that were used are provided in supplementary material). We generated data with three levels of missing data (0, 5, and 30%) and four sample sizes: 100, 250, 500, and 1000. We repeated our experiments 50 times. Performance was measured by the PAG accuracy measure that evaluates how many edges were oriented correctly in the output PAG compared with the ground-truth PAG (Fig. 1b). We also estimated the correctness of the skeleton by calculating precision and recall metrics, where the former estimates the number of edges inferred correctly to the total number of inferred edges and the latter estimates the number of edges inferred correctly to the number of edges in the ground-truth graph (Fig. 1).

**Fig. 2:**
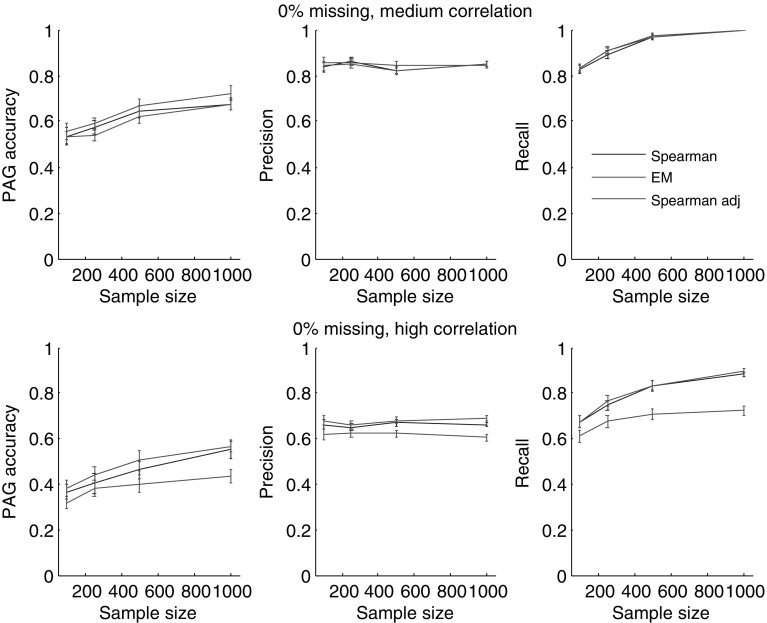
The accuracy of the BCCD algorithm (PAG accuracy, precision, and recall) for the Waste Incinerator Network for data with medium and high correlation when there are no missing values

We investigate the effect of different approaches to estimate the correlation matrix (described in Steps 1 and 2 in Sect. 4) on the accuracy of the causal discovery algorithm. We consider the following alternatives:

1. Pearson correlation with EM. (EM)
2. Spearman correlation with mean imputation. (Spearman mean)
3. Spearman correlation with list-wise deletion. (Spearman list-wise)
4. Pairwise Spearman correlation. In this approach, we *do not* make an adjustment of the Spearman correlation based on (4). (Spearman not adjusted)
5. Pairwise Spearman correlation with adjustment. In this approach, we *do* make an adjustment of the Spearman correlation based on (4). (Spearman pairwise)

If the obtained matrix is not positive definite, it is projected to the closest positive definite matrix [24]. We repeat these tests for two different causal discovery algorithms: BCCD and PC.

When there is no missing data, Spearman mean, Spearman list-wise, and Spearman pairwise provide the same results. Thus, we compare only three alternatives: EM, Spearman, Spearman adjusted. Figure 2 represents the results of BCCD for two cases: when the data has a medium correlation and high correlation. For medium correlation, Spearman adjusted performs similarly with the other two methods, but for high correlation it performs significantly worse than Spearman not adjusted and EM. The factor that is causing this difference is the ill-defined determinant of the correlation matrix which is close to zero when the correlation is high. Adjustment of the correlation matrix using (4) increases the correlations even more, which results in a non-positive definite correlation matrix and loss of conditional independencies between variables compressed in the correlation matrix. This results in many incorrect edges and a low PAG accuracy. Thus, when the correlation between variables is high, adjusting the Spearman correlation may lead to significantly worse results. Based on this conclusion, we did not consider Spearman adjusted for tests with missing values, since it already showed significantly worse performance compared to Spearman not adjusted.

**Fig. 3:**
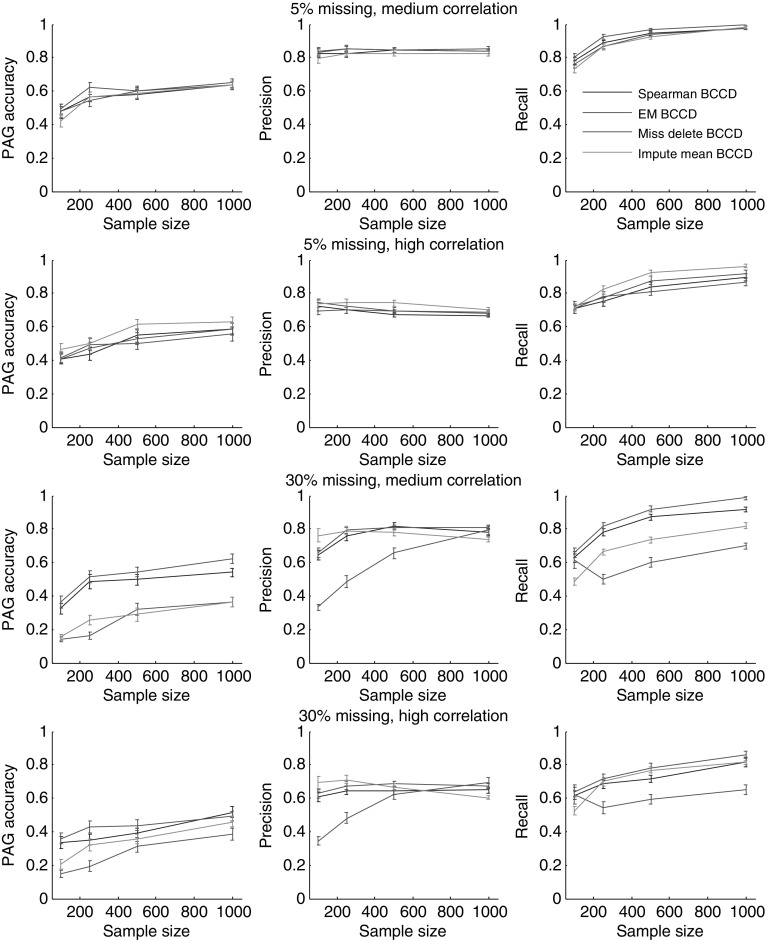
The accuracy of the BCCD algorithm (PAG accuracy, precision, and recall) for the Waste Incinerator Network for data with medium and high correlation at two levels of missing values: 5% missing, 30% missing

Figure 3 shows the results of BCCD when the data have a low (5%) and high (30%) percentage of missing values. When percentage of missing values is low (5%) the differences between EM, Spearman mean, Spearman list-wise, and Spearman pairwise are not significant. When the percentage of missing values is high (30%), EM performs significantly better than Spearman for both medium and high correlation. One of the main factors that leads to this difference in performance between EM and pairwise correlation is a non-positive definite correlation matrix with a high number of missing values. The advantage of the EM algorithm in that case is that it outputs a positive definite matrix. Even though we projected the Spearman correlation matrix to a positive definite correlation matrix, simulation tests show that EM provides more accurate results. When the percentage of missing values is high, mean imputation leads to a decrease in variance which results in lower accuracy. As expected, Spearman list-wise performs worse than all other methods due to significant loss of information when estimating the correlation when the amount of missing data are high.

**Fig. 4:**
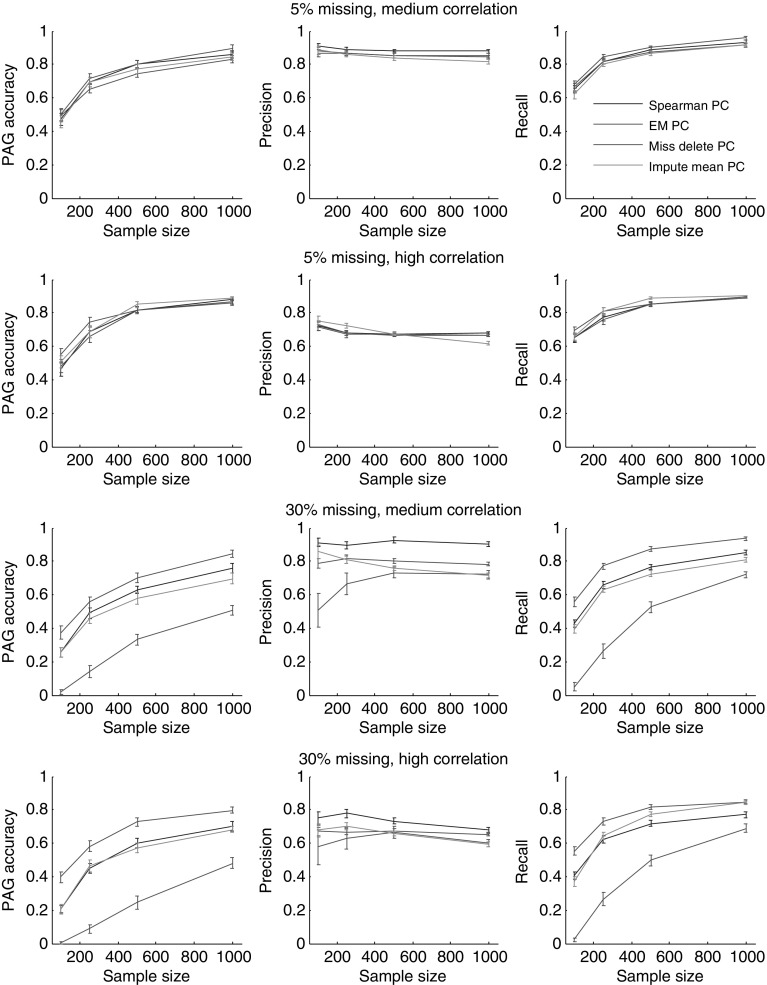
The accuracy of the PC algorithm (PAG accuracy, precision, and recall) for the Waste Incinerator Network for data with medium and high correlation at two levels of missing values: 5% missing, 30% missing

We repeated the same experiments with PC and obtained similar patterns, see Fig. 4. When 5% of the data is missing, no significant difference between the methods is observed. When 30% of the data is missing, EM gives significantly better PAG accuracy. Although BCCD is a more advanced algorithm than PC, it provides lower PAG accuracy in these experiments. It happens because PC infers the directions based on the assumption that there are no unobserved common causes and no selection bias, while BCCD does not rely on these assumptions. Since waste incinerator network does not contain unobserved common causes and selection, PC can infer the correct structure of the network more easily than BCCD. For both BCCD and PC, increasing the sample size improves recall and PAG accuracy, while it does not help to improve the precision. When sample size becomes large, our method starts to detect more spurious edges leading to a decrease in precision in the simulation studies. An increase in the number of spurious edges with an increase in sample size is a common problem in structure learning, since with a high sample size even very small correlations between variables become significant. In this case, we are not talking about ’spurious’ correlations (which would be resolved with more data), but about real but weak correlations that are often present in complex, real-world systems, but that are overlooked (not detected) in small data sets.

We compare our results with the results obtained for undirected graphical models in Ref. [1, 53]. The two main results for undirected graphical models are: (1) Spearman and EM both perform well, while EM performs slightly better. (2) Making the projection for the correlation matrix to the closest positive definite matrix improves the results. The main results that we obtained for directed graphical models are: (1) EM performs *significantly* better than Spearman with projection for data with a high percentage of missing values and a high correlation between variables. (2) Working with directed graphical models one should be careful in applying the adjustment of the Spearman correlation. This adjustment may destroy the positive definiteness property of the matrix even when there are no missing values in the data. The difference in results between undirected and directed graphical models can arise because undirected graphical models are typically inferred under sparseness constraints. Optimizing the correlation matrix under sparseness constraints decreases the number of spurious dependencies that might otherwise arise due to an ill conditioned or even non-positive definite correlation matrix. We do not have a similar type of regularization to estimate the mutual information in (5) and (6), which then may explain the larger difference in performance between EM, Spearman, and Spearman adjusted.

**Fig. 5:**
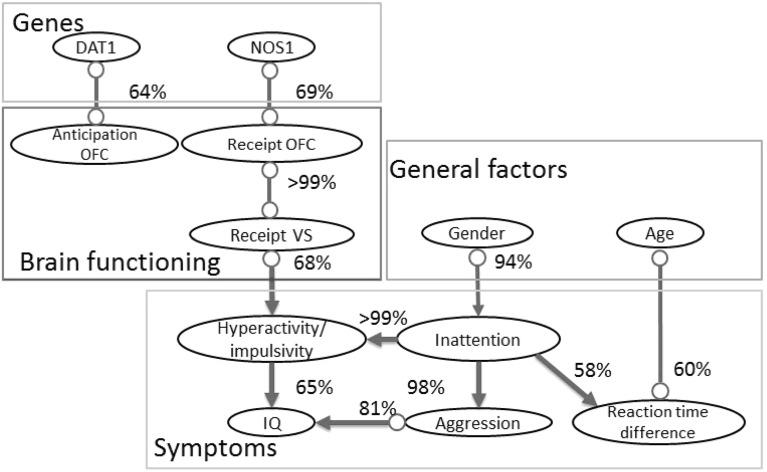
The causal graph representing causal relationships between variables for the MID task ADHD data set. The graph represents a PAG, where edge directions are marked with “ − ” and “>” for invariant edge directions and with “$\circ$” for non-invariant edge directions. The reliability of an edge between two variables is depicted with a percentage value near each edge

### ADHD data

We have applied the BCCD algorithm with EM to two data sets representing two different ADHD studies performed as a part of the NeuroIMAGE study.

#### MID tasks study

The first study [51] investigated the brain response during reward anticipation and receipt with a monetary incentive delay (MID) task in a large sample of adolescents and young adults with ADHD, their unaffected siblings and healthy controls. All subjects participated in cognitive testing and neuroimaging. The brain activation was measured in ventral striatum (VS) and orbital-frontal cortex (OFC) brain areas during the reward anticipation and receipt [52]. The data set contained 409 participants: 189 probands with ADHD, 104 unaffected siblings, and 116 age-matched controls. Since the presence of the unaffected siblings can blur the effect of the genes, we did not include them in our study and consider only ADHD patients and healthy controls. Approximately 10% of data is missing for this study. The main reason for the presence of missing values in this data set was that part of the experiments was very time-consuming and as a result not all the results were available yet, leading to missing values in the data set. Thus, we may assume for this data set that data are missing completely at random. Scatter plots did not reveal any non-monotonic dependencies, supporting our hypothesis of monotonic dependencies.

Using BCCD, we wanted to infer the endophenotipic model [19] that explains the relationships between genes, brain functioning, behaviors, and disease symptoms. To apply causal discovery to this data set, domain experts selected 12 variables. These variables include general characteristics, genetic factors, comorbid disorders, symptoms, and results of the MID task experiments:

1. Gender (male/female).
2. Age.
3. IQ.
4. DAT1 risk gene (present/not present).
5. NOS1 risk gene (present/not present).
6. Inattention symptoms (score assessed by KSADS and CPRS-R:L).
7. Hyperactivity/impulsivity symptoms (score assessed by KSADS and CPRS-R:L).
8. Aggression [(presence/absence of Oppositional Defiant Disorder (ODD) or Conduct disorder (CD)].
9. Brain activation in OFC during receipt (Receipt OFC).
10. Brain activation in VS during receipt (Receipt VS).
11. Brain activation in OFC during anticipation (Anticipation OFC).
12. Reaction time difference (the difference in reaction time with and without a reward).

**Fig. 6:**
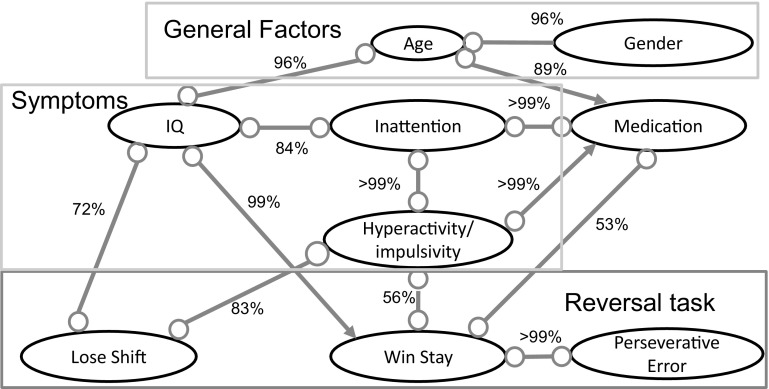
The causal graph representing causal relationships between variables for the reversal task ADHD data set. The graph represents a PAG, where edge directions are marked with “−” and “>” for invariant edge directions and with “$\circ$” for non-invariant edge directions. The reliability of an edge between two variables is depicted with a percentage value near each edge

The initial data set contained two different estimates of the ADHD symptoms: one estimated by parents and another one estimated by a psychiatrist. Since these are highly correlated, it makes no sense to include both. We decided to keep the parent scores, because an initial analysis revealed slightly more variation and slightly stronger correlation with the other variables. These symptom scores represent the quantiles in the population adjusted by age and gender. We readjusted these scores to be able to see the explicit effect of gender.

Partially due to the small sample size, the BCCD algorithm inferred only the skeleton of the network, but not the direction of the edges for the resulting network. However, including prior knowledge about the domain that no variable in the network can cause gender, and the endophenotypic assumption from Ref. [19] that symptoms are the consequence of problems with brain functioning, BCCD inferred the direction of several edges.

The causal network learned from the data is presented in Fig. 5. The figure indicates network edges with an estimated link of 50% or above. The resulting network structure provides an endophenotypic model that connects genes, brain functioning, and symptoms together. The causal model suggests association of genes with brain activation during the monetary incentive delay task. This model confirms several causal pathways that were previously presented in other studies and suggests new endophenotypic pathways.

Our causal model suggests that NOS1 is associated with brain activation in OFC during reward receipt and DAT1 with brain activation during reward anticipation. The effect of genes on brain functioning was also claimed in other studies [16, 27]. The model proposes that the reaction time depends on the age of the subject and his/her level of inattention. In Ref. [25], a similar conclusion was drawn about the increase in reaction time up to early adulthood. The level of inattention symptoms depends on the gender of the subject. This statement is confirmed by different studies in the field of ADHD [5]. The level of hyperactivity/impulsivity depends on the level of inattention and on the problems with brain activation in MID task in VS. The effect of inattention on hyperactivity/impulsivity was also found in Ref. [54]. The level of aggression is associated with the level of IQ and inattention level.

Most studies focus on association between symptoms and reward *anticipation* rather than between symptoms and reward *receipt* and several studies report a link between these two variables [40, 45], whereas others do not [37]. The causal model inferred in our study suggests a causal path from reward *receipt* to hyperactivity/impulsivity symptoms and no clear link between reward *anticipation* and symptoms. Moreover, the causal model provides computational evidence for new causal association between genes, brain functioning, and symptoms, from NOS1 to hyperactivity/impulsivity symptoms through brain functioning during receipt. The model inferred in this study should be treated with care, but can suggest further studies, zooming in on some of the pathways found through this analysis.

#### Reversal task study

The second study investigated the behavioral response during a probabilistic reversal learning task (PRL). With the PRL, one can learn whether participants are able to adapt to a changing situation, whether they are able to learn a (new) rule, and possibly whether participants are sensitive to reward and punishment. The participants of the reversal task study partially overlap with the participants from the MID task study. However, since the MID task experiments were performed several years before the reversal task study, in the reversal task study the participants are older.

We applied BCCD to investigate the relationships between ADHD symptoms and problems with reversal behavior. Based on the domain knowledge experts selected nine variables that are associated with ADHD and may influence the outcome of the reversal task:

1. Gender (male/female).
2. Age.
3. IQ.
4. Inattention symptoms (score assessed by KSADS and CPRS-R:L).
5. Hyperactivity/impulsivity symptoms (score assessed by KSADS and CPRS-R:L).
6. Win-stay score (percentage of trials in which participants chose the same stimulus after a win).
7. Lose-shift score (percentage of trials in which participants chose the other stimulus after a loss).
8. Preservative error score (the amount of errors made after reversal that were related to picking the previous stimulus).
9. Medication status (naive/not naive).

To infer a more accurate causal network, we included in the model the prior knowledge that nothing can cause gender.

The causal network inferred by BCCD is presented in Fig. 6. This network suggests the effect of age on subject’s IQ and whether the medication was prescribed or not. Moreover, age is associated with gender in this model, which happens due to age/gender unbalance in the sample. In contrast to the causal model in the MID task (Fig. 5), this causal model does not find any link between gender and symptoms. A possible explanation can be the observation [29] that gender unbalance vanishes when ADHD patients get older and become adults. Since in the reversal task study subjects are approximately 3.6 years older than in the MID task study, this might explain why in reversal task study there is no effect of gender on symptoms.

Analysis of the causal links between symptoms PRL experiment outcomes suggest that IQ and hyperactivity/impulsivity are associated with variables related to reversal learning. Subjects with a lower IQ and higher level of hyperactivity/impulsivity have a higher percentage of lose-shift responses and a lower percentage of win-stay responses, suggesting sensitivity for punishment but not for reward in participants with more hyperactivity/impulsivity symptoms. Although we did not find a direct association between symptoms and age, older participants with ADHD tend to have less hyperactivity/impulsivity symptoms than younger participants [18], possibly relating age to performance in the PRL. Probably a sample with higher age differences is needed to be able to infer such a pattern from the data.

The association of IQ with both win-stay and lose-shift may be related to the difficulty of the task in general. Participants with a lower IQ have more problems with performing the task but are not specifically more sensitive to punishment than reward. Additionally, with the PRL one can investigate how well people can adapt to a changing rule, which may be difficult for subjects with ADHD [2]. Although this is the first study of causal analysis with ADHD and PRL performance, it shows promising possibilities for future research.

## Discussion and conclusions

The simulation study shows that the EM algorithm performs better than Spearman with pairwise correlation, mean imputation, and list-wise deletion for directed graphical models when the percentage of missing values is high, while providing similar results when the percentage is low. Comparing EM with pairwise Spearman, these results can be explained by the fact that the correlation matrix can become non-positive definite when calculating pairwise correlation with missing values. This leads to an incorrect estimate of the determinant. Estimation of the correlation matrix using the EM algorithm outputs a positive definite matrix that results in a better accuracy of the algorithm. Thus the EM with Gaussian transformation proposed in this paper performs better than the Spearman pairwise correlation method proposed in Ref. [53] for causal discovery. EM outperforms mean imputation due to a more sophisticated method to impute missing values that does not reduce variance. Bad performance of list-wise deletion when the percentage of missing values is high is logical, since main part of the data is not used when applying this method. A simulation study using the PC algorithm instead of BCCD confirmed these results. Although the EM algorithm is computationally more expensive than alternative methods described in the paper such as pairwise correlation, it should be calculated only once. For a data set of 15 variables, it does not take longer than a minute.

Where pairwise and list-wise deletion correlation estimation that rely on the assumption that data are missing completely at random (MCAR), EM assumes that data are “just” missing at random (MAR). This assumption applies more often in practice and thus increases the range of data sets for which it can be used.

The simulation study also shows that adjustment of the Spearman correlation when the correlation is high can decrease the accuracy of the causal discovery algorithm. The determinant of the correlation matrix is close to zero when the correlation between variables is high. When applying the adjustment of the correlation matrix, the correlation increases even more which can again result in a non-positive defined matrix determinant. For medium correlation, Spearman adjusted and Spearman not adjusted show similar accuracy. Thus, we can conclude that for estimating mutual information it is better not to adjust the Spearman correlation.

Using the BCCD algorithm, we inferred an endophenotypic model of ADHD during the MID task. The resulting model explains the effect of genes on brain functioning, the effect of brain functioning and general factors on disease symptoms, and an interaction between these symptoms. This model confirms previous findings in the literature and proposes new causal links between variables. The model shows evidence for receipt and against anticipation endophenotypes and highlights the need to extend genetic research on this less expected endophenotype. In this sense, this study suggests promising new pathways for genetic research in ADHD that need to be confirmed by genetic imaging studies.

BCCD inferred a model explaining the interaction between symptoms and problems with reversal learning measured during the PRL task. This model suggests that the main factors that influenced the outcome of the experiments were hyperactivity/impulsivity, IQ, and medication. These results provide a new insight into the reversal learning problems and can improve its treatment.

## Electronic supplementary material

Below is the link to the electronic supplementary material.

Supplementary material 1 (pdf 99 KB)
