# Supplementary material for: Handling hybrid and missing data in constraint-based causal discovery to study the etiology of ADHD
Source: Int J Data Sci Anal. 2016 Dec 2;3(2):105–19. doi: 10.1007/s41060-016-0034-x (PMC5479362; doi:10.1007/s41060-016-0034-x)
Supplement: Supplementary file 1 — Supplementary material 1 (pdf 99 KB) [file 41060_2016_34_MOESM1_ESM.pdf]

Noname manuscript No.
(will be inserted by the editor)

Handling hybrid and missing data in constraint-based causal discovery to study the etiology of ADHD

Elena Sokolova[1], Daniel von Rhein[2], Jilly Naaijen[2], Perry Groot[1], Tom Claassen[1], Jan Buitelaar[2] and Tom Heskes[1]

Received: date / Accepted: date

Supplementary material

Parameters for the Waste Incinerator Network when the correlation between variables is extreme-high:

$P(B = \mathit{stable}) = 0.15$
$P(F = \mathit{intact}) = 0.05$
$P(W = \mathit{household}) = 2/7$
$P(E|\mathit{intact}, \mathit{industrial}) = \mathcal{N}(-3.9, 0.2)$
$P(E|\mathit{intact}, \mathit{household}) = \mathcal{N}(-3.2, 0.2)$
$P(E|\mathit{defective}, \mathit{industrial}) = \mathcal{N}(-0.40.1)$
$P(E|\mathit{defective}, \mathit{household}) = \mathcal{N}(-0.5, 0.1)$
$P(D|\mathit{stable}, \mathit{industrial}, e) = \mathcal{N}(6.5 + e, 0.03)$
$P(D|\mathit{stable}, \mathit{household}, e) = \mathcal{N}(6.0 + e, 0.04)$
$P(D|\mathit{unstable}, \mathit{industrial}, e) = \mathcal{N}(7.5 + e, 0.1)$
$P(D|\mathit{unstable}, \mathit{household}, e) = \mathcal{N}(7.0 + e, 0.1)$
$P(C|\mathit{stable}) = \mathcal{N}(-2, 0.3)$
$P(C|\mathit{unstable}) = \mathcal{N}(-1, 0.5)$
$P(L|D) = \mathcal{N}(3 - 0.5d, 0.25)$
$P(MW|\mathit{industrial}) = \mathcal{N}(0.5, 0.01)$
$P(MW|\mathit{household}) = \mathcal{N}(-0.5, 0.005)$
$P(ME, d, mw) = \mathcal{N}(d + mw, 0.002)$

[1]Faculty of Science, Radboud University Nijmegen, The Netherlands
[2]Donders Institute for Brain, Cognition and Behaviour, Radboud University Medical Center Nijmegen, The Netherlands

Parameters for the Waste Incinerator Network when the correlation between variables is medium:

$P(B = \mathit{stable}) = 0.15$
$P(F = \mathit{intact}) = 0.05$
$P(W = \mathit{household}) = 2/7$
$P(E|\mathit{intact}, \mathit{industrial}) = \mathcal{N}(-2, 0.2)$
$P(E|\mathit{intact}, \mathit{household}) = \mathcal{N}(-1.8, 0.2)$
$P(E|\mathit{defective}, \mathit{industrial}) = \mathcal{N}(-0.40.1)$
$P(E|\mathit{defective}, \mathit{household}) = \mathcal{N}(-0.5, 0.1)$
$P(D|\mathit{stable}, \mathit{industrial}, e) = \mathcal{N}(6.5 + e, 0.3)$
$P(D|\mathit{stable}, \mathit{household}, e) = \mathcal{N}(6.0 + e, 0.4)$
$P(D|\mathit{unstable}, \mathit{industrial}, e) = \mathcal{N}(7.5 + e, 0.1)$
$P(D|\mathit{unstable}, \mathit{household}, e) = \mathcal{N}(7.0 + e, 0.1)$
$P(C|\mathit{stable}) = \mathcal{N}(-2, 0.3)$
$P(C|\mathit{unstable}) = \mathcal{N}(-1, 0.5)$
$P(L|D) = \mathcal{N}(3 - 0.5d, 0.25)$
$P(MW|\mathit{industrial}) = \mathcal{N}(0.5, 0.01)$
$P(MW|\mathit{household}) = \mathcal{N}(-0.5, 0.05)$
$P(ME, d, mw) = \mathcal{N}(d + mw, 0.2)$
